# Non-Uniform Concentric Rings Design for Ultra-Wideband Arrays

**DOI:** 10.3390/s19102262

**Published:** 2019-05-16

**Authors:** Marco A. Panduro, Alberto Reyna, David H. Covarrubias

**Affiliations:** 1 CICESE Research Center, Electronics and Telecommunications Department, Carretera Ensenada-Tijuana No. 3918, Zona Playitas, Ensenada, Baja California 22860, Mexico; dacoro@cicese.mx; 2 Unidad Académica Multidisciplinaria Reynosa-Rodhe, Universidad Autónoma de Tamaulipas (UAT) Carretera Reynosa-San Fernando, Reynosa, Tamaulipas 88779, Mexico; alberto.reyna@uat.edu.mx

**Keywords:** ultra-wideband, aperiodic antenna arrays, concentric rings geometry, side lobe level

## Abstract

This paper presents the design of aperiodic concentric ring arrays for ultra-wide bandwidths (UW-ACRA). This design of ultra-wideband arrays considers the synthesis of concentric rings in two cases: 1) non-uniform spacing between rings with non-uniform spacing between antenna elements of the same ring (UW-ACRA*_elements_*); and 2) non-uniform spacing between rings assuming that spacing between antenna elements of the same ring to be equal (UW-ACRA*_rings_*). This is in order to eliminate the occurrence of grating lobes and generating array structures with useful ultra-wideband properties. The synthesis process is carried out by the well-known method of differential evolution (DE). Wireless sensor networks can take advantage of these properties to achieve less data traffic, efficient delivery of information and better energy efficiency.

## 1. Introduction

There is a great interest for designing antenna arrays in wireless sensor networks [1]. This is because wireless sensor networks can take advantage of the properties of antenna arrays that facilitate less data traffic, efficient delivery of information and better energy efficiency [1]. Non-uniform or aperiodic [2,3] antenna arrays provide several advantages with respect to traditional periodic arrays. They present low side lobe level (*SLL*) (no grating lobes) over arbitrarily large bandwidths [4], require significantly fewer elements to generate a desired beam shaped [4], and have the ability to achieve a low *SLL* without requiring any amplitude tapering [5]. Approaches to achieve low relative *SLL* with periodic arrays often require significant amplitude tapering and are therefore rather inefficient. 

The work of Werner et al. highlights in the previous work of wideband aperiodic antenna arrays [2,3,6,7,8]. Werner et al. have analyzed different geometries and array configurations with different optimization techniques, such as polyfractal arrays [3,6], antenna arrays based on power series representations [7], planar antenna arrays based on aperiodic tilings [8], planar array layouts exploiting rotational symmetry [2] and aperiodic antenna arrays with an evolutionary strategy [4], among others. These previous papers have illustrated that the design of planar aperiodic antenna arrays can yield very useful ultra-wideband properties. 

Although there are several works on the cutting edge of aperiodic circular [9], concentric rings [10,11], or planar arrays [2,3,4], a performance evaluation dealing with non-uniform concentric rings arrays is lacking for ultra-wide bandwidths. Therefore, the aim of this paper is to illustrate the design of non-uniform antenna arrays using the geometry of concentric rings for ultra-wideband performance. As the inter-element spacing increases beyond the limit of a wavelength at the operating frequency, the radiation performance deteriorates due to the arising of grating lobes [12]. However, when each radiating element is arranged within a concentric circular lattice, the appearance of grating lobes can be mitigated and controlled. By exploiting this intrinsic property of the concentric ring array layout, an optimization procedure can be employed to synthesize several planar arrays with controlled *SLL* over a wide frequency range. The frequency range is specified using the fractional bandwidth (*FBW*) defined previously in the literature [13]. The array bandwidth is determined by the minimum element spacing. Element spacing is typically constrained to 0.5*λ* at the lowest operating frequency of the array to avoid overlapping elements and undesirably large mutual coupling [2,3,4]. For arrays able to operate effectively with no grating lobes and low side lobes at a minimum element spacing of *bλ* (where *b* ≥ 0.5), the resulting frequency bandwidth is then 2*b*:1 [2,3,4].

This design of ultra-wideband arrays considers the synthesis of concentric rings in two cases: (1) aperiodic array with non-uniform spacing between rings and between antenna elements of the same ring (UW-ACRA*_elements_* case); and (2) aperiodic array with non-uniform spacing between rings assuming that spacing between antenna elements of the same ring to be equal (UW-ACRA*_rings_* case). 

The innovative contribution of this paper is the application of an evolutionary optimization algorithm to design non-uniform concentric rings arrays with desirable radiation characteristics for wideband performance. This wideband performance is determined by the effect of varying the minimum element spacing for different configurations of aperiodic concentric ring arrays.

The synthesis aims to eliminate the occurrence of grating lobes and generating array structures with useful ultra-wideband properties. The synthesis process is carried out by the method of differential evolution (DE) [14]. 

The remainder of the paper is organized as follows. Section 2 states the design problem and describes the optimization procedure employed. Section 3 presents and discusses the simulation results. Finally, the summary and conclusions of this work are presented in Section 4.

## 2. Problem Statement

### 2.1. Array Factor Model

**Figure 1:**
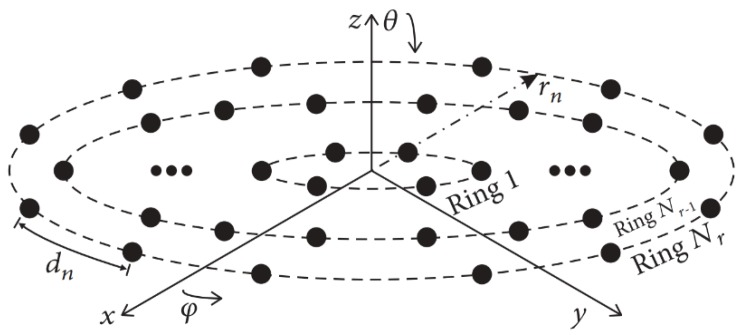
Non-uniform concentric ring array.

Among the possible planar array configurations, the concentric ring array exhibits the interesting properties of a nearly invariant pattern for a full azimuthal coverage and main beam symmetry [12]. The array factor for this geometry can be determined using the array factor expression for a planar antenna array. The concentric ring array consists of *N_r_* rings and *N_e_* antenna elements in each ring on the *x*-*y* plane, as shown in Figure 1. The array factor of this geometry can be determined using the next expression [15]:

(1) $$AF\left(\theta ,\phi \right)=\sum_{n=1}^{N_{r}}\sum_{m=1}^{N_{e}}exp\left[jk\left(x_{nm}\left(u\right)+y_{nm}\left(v\right)\right)\right],$$

where *u* = sin*θ*cos*ϕ*, *v* = sin*θ*sin*ϕ*, *k* = 2π/*λ* is the phase constant with *λ* representing the operating wavelength, *θ* is the angle of a plane wave in the elevation plane and *ϕ* is the angle of a plane wave in the azimuth plane. In this case, the position of each antenna element is defined by (*x_nm_* = *r_n_*cos*φ_nm_*, *y_nm_* = *r_n_*sin*φ_nm_*), where *r_n_* represents the radial distance of each ring from the common center of the array until the nth ring. The antenna element distribution for the case of (UW-ACRA*_rings_*) is given in each circular ring by *φ_nm_* = 2π(*m* − 1)/*Ne*. This design case of concentric ring array does not consider a central element in the origin.

The array factor as a function of the non-uniform inter-element spacing in each ring and the different radius of each ring, that is, the UW-ACRA*_elements_* case [11,16,17,18], can be determined using the next expression:

(2) $$AF\left(\theta ,\phi ,d,r\right)=\sum_{n=1}^{N_{r}}\sum_{m=1}^{N_{e}}exp\left[jkr_{n}\left(u\cos\varphi_{nm}+v\sin\varphi_{nm}\right)\right],$$

where **d** = [*d*_1,1_, *d*_1,2_, …, *d*_1*,N*1_*; d*_2,1_, *d*_2,2_,…, *d*_2*,N*2_; …; *d_Nr_*_,1_, *d_Nr_*_,2_,…, *d_Nr,Ne_*] *d_nm_* represents the arc longitude from element *m* to element *m* + 1 on the *n*th ring of the array. The radii of the antenna array are grouped in ***r*** = [*r_1_, r_2_, …, r_n_, …, r_Nr_*]. Equation (2) is a function of the product of the radius and the phase constant, i.e., *kr_n_* = 2π*r_n_*/*λ*. This gives the perimeter of each ring (in terms of *λ*) which can be calculated as the sum of all arc longitudes or separations between antenna elements, so, *kr_n_* = 2π*r_n_*/*λ* =$\;\sum_{m=1}^{e}d_{m}\left(\lambda \right),\;\forall n\in N_{r}$. The notation in *d_m_* (*λ*) denotes that the separations between antenna elements are in terms of wavelength.

The frequency range is specified by using the fractional bandwidth (*FBW*) defined as [13]:

(3) $$FBW=\frac{f_{U}-f_{L}}{f_{M}}$$

where *f_M_*, *f_U_* and *f_L_* are respectively the center, the upper and the lower frequency. According to (3), arrays are considered wideband when 0.2 < *FBW* < 0.5 and ultrawideband when *FBW* > 0.5 [13].

This design problem of an ultra-wideband antenna array consists of minimizing the peak sidelobe level (*PSLL*) of the array factor *AF*(*θ*,*ϕ*,**d**,*r*) [2]:

(4) PSLL=max(AF(θ,ϕ,d,r)∉Mainbeam)

while enforcing a minimum element spacing (*d_min_*), i.e., with respect to a minimum element spacing generated (*d_generated_*) by the concentric ring structure

(5) $$d_{min}\geq d_{generated}$$

which determines the bandwidth of the concentric ring array. Element spacing is typically constrained to 0.5*λ* at the lowest operating frequency of the array to avoid overlapping elements and undesirably large mutual coupling [2]. For arrays able to operate effectively with no grating lobes and low side lobes at a minimum element spacing of *bλ* (where *b* ≥ 0.5), the resulting frequency bandwidth is then 2*b*:1 [2]. Therefore, the design problem can be set as the minimization of the next objective function (*OF*):

(6) $$OF=PSLL+abs\left(d_{min}-d_{generated}\right)$$

### 2.2. Optimization Procedure

**Figure 2:**
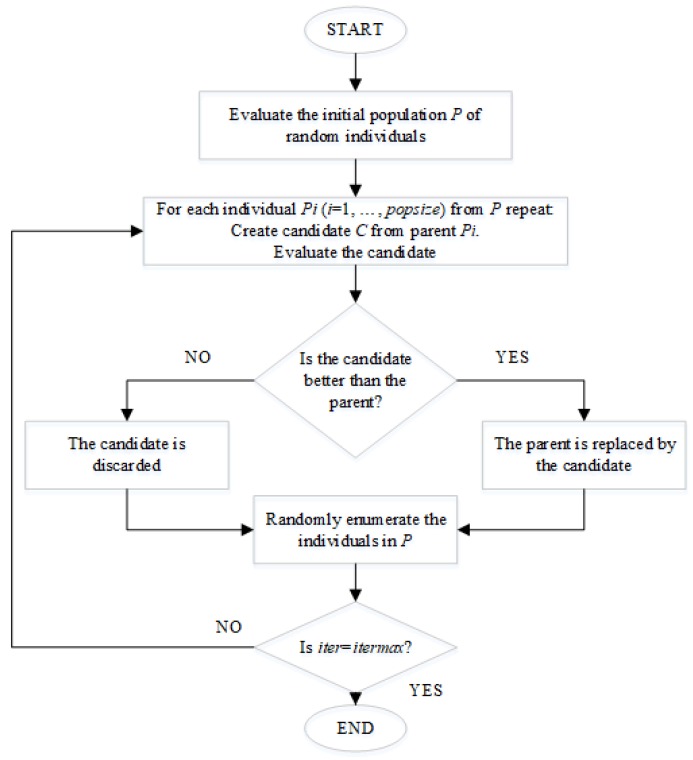
Flowchart of the differential evolution (DE) optimization applied [24].

The optimization procedure is carried out by Differential Evolution due to its effectiveness solving antenna array designs [19,20,21,22,23]. The flowchart for the DE optimization procedure is shown in Figure 2 [24]. The initial population is randomly generated. Each member (or potential solution) of the population is represented by $x_{i,G}$ for i=1, 2, 3, …, PopSize. Then, for each generated vector $\;x_{i,G},$ a mutation vector (*v_i_*,*_G_*_+1_) is calculated in according to [14]:

(7) $$v_{i,G+1}=x_{r1,G}+F\cdot \left(x_{r2,G}-\;x_{r3,G}\right)$$

The indexes $r_{1},r_{2},\;r_{3}\in \left\{1,\;2,\;\ldots ,\;PopSize\right\}$ are different among them ($r_{1}\neq r_{2}\neq r_{3})$ and also different from index i. PopSize is the size of the population. F is a real parameter ∈[0, 2], which controls the amplification on the differential variation $\left(x_{r2,G}-\;x_{r3,G}\right)$.

Once the stage of mutation is finished, the mutation vector undergoes for crossover operation. The trial vector $u_{i,G+1}=\left(u_{1i,G+1},\;u_{2i,G+1},\;\ldots ,\;u_{Di,G+1}\right)$ is performed in according to [13]:

(8) $$u_{ji,G+1}=\{\begin{array}{c} v_{ji,G+1}\;if\;\left(randb\left(j\right)\leq CR\right)or\;j=rnbr\left(i\right)\\ x_{ji,G}\;if\;\left(randb\left(j\right)>CR\right)and\;j\neq rnbr\left(i\right) \end{array}$$

In (8), j=1, 2, …, D,  randb(j) is a random number  ∈[0, 1], CR∈[0,1] is the crossover ratio, rnbr(i) is randomly chosen index ∈1, 2, …, D (*D* is the dimension of the problem) which ensures that $u_{i,G+1}$ gets at least one parameter from $v_{i,G+1}$.

Each solution vector is codified by the values of spacing between rings and the values of spacing between the elements on the *n*th ring. The fitness evaluation for the array factor of each solution vector must follow the considerations given for Equation (6).

To decide whether the trial vector $u_{i,G+1}$ should be selected for the next generation or not, is compared to the target vector $x_{i,G}$ by greedy criterion. If the trial vector $u_{i,G+1}$ has got smaller fitness value than $x_{i,G}$, then $x_{i,G+1}$ is set to $u_{i,G+1}$; otherwise, the old value of $x_{i,G}$ is retained. This procedure is repeated over and over until the algorithm reaches the maximum number of generations *G*. Detailed information of the DE optimization procedure can be found in [14]. 

## 3. Simulation Results

The method of DE was implemented in Matlab following the methodology described in [12]. Four array configurations are considered for both design cases: 1) *N_T_* = 90 antenna elements distributed in *N_r_* = 5 rings, 2) *N_T_* = 126 with *N_r_* = 6, 3) *N_T_* = 168 with *N_r_* = 7 and 4) *N_T_* = 216 with *N_r_* = 8, for *N*_1_ = 6, *N*_2_ = 12, *N*_3_ = 18, *N*_4_ = 24, *N*_5_ = 30, *N*_6_ = 36, *N*_7_ = 42 and *N*_8_ = 48. The ultra-wideband characteristics of these aperiodic concentric ring antenna arrays have been analyzed, focusing on *PSLL* performance at minimum element spacing ranging from 0.5*λ* to 10*λ*. This minimum spacing range corresponds to operating frequencies of *f*_0_ to 20*f*_0_, with *f*_0_ designated as the lowest operating frequency of the array.

**Figure 3:**
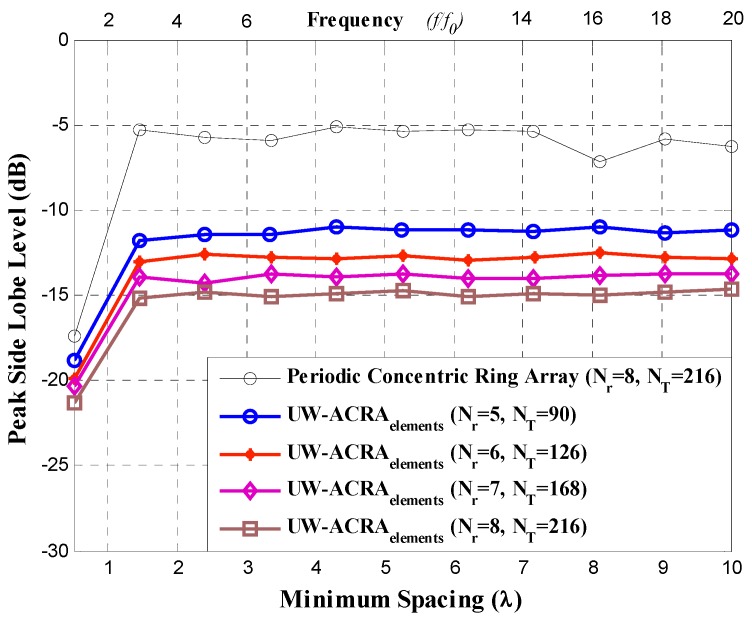
Peak sidelobe level (*PSLL*) performance of the aperiodic concentric rings array (UW-ACRA*_elements_*).

**Figure 4:**
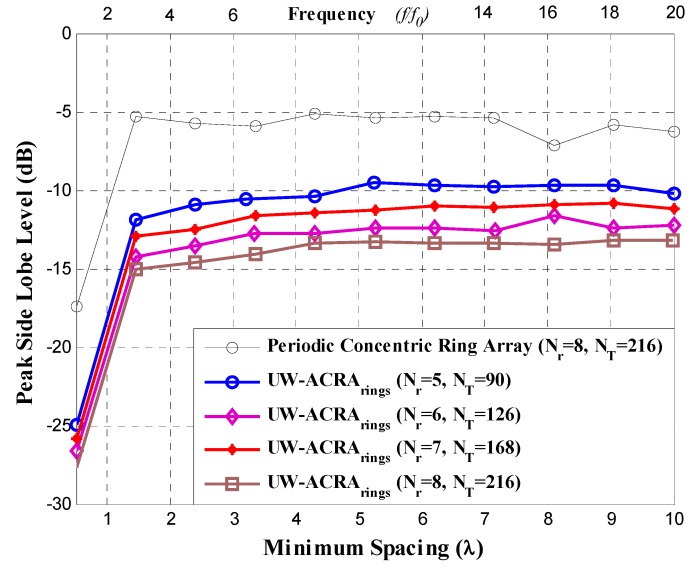
*PSLL* performance of the aperiodic concentric rings array (UW-ACRA*_rings_*).

**Figure 5:**
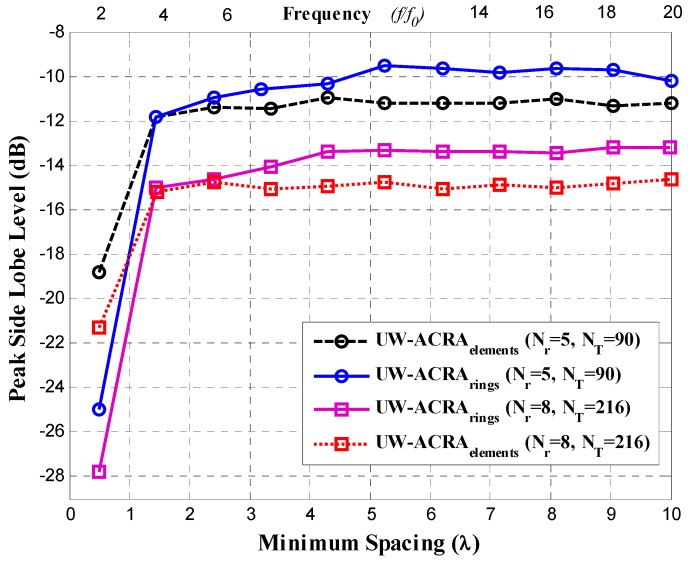
Comparison of the *PSLL* performance between the case of UW-ACRA*_elements_* and the case UW-ACRA*_rings_*.

Figure 3 and Figure 4 illustrate the *PSLL* performance of the non-uniform concentric ring arrays optimized by DE for the case of UW-ACRA*_elements_* and UW-ACRA*_rings_*, respectively. The optimization of both geometries provides low values of *PSLL* for the element spacing corresponding to frequencies of *f*_0_ to 20*f*_0_. The behavior of the periodic concentric ring array is determined using *N_T_* = 216 antenna elements with *N_r_* = 8 rings. Both aperiodic geometries present a better *PSLL* performance with respect to the periodic case. The lowest values of *PSLL* (in each design case) are found for *d_min_* = 0.5*λ* with lower values for UW-ACRA*_rings_* (ranging from −24.95 dB to −27.82 dB) with respect to UW-ACRA*_elements_* (from −18.84 dB to −21.30 dB). If the minimum spacing is increased (operating frequencies greater than 3*f*_0_) the *PSLL* performance deteriorates for periodic case, and the case of UW-ACRA*_elements_* presents better *PSLL* values with respect to UW-ACRA*_rings_*, as shown in Figure 5. The minimum and maximum values of *PSLL* found by the case of UW-ACRA*_elements_* and UW-ACRA*_rings_* are *PSLL_max_* = −10.96 dB, *PSLL_min_* = −15.92, and *PSLL_max_* = −9.55 dB and *PSLL_min_* = −15.01, respectively, for the element spacing corresponding to operating frequencies greater than 3*f*_0_. Furthermore, the case of UW-ACRA*_elements_* covers totally the UW-ACRA*_rings_* for frequency values greater than 5*f*_0_ reaching the maximum difference in *d_min_* = 5.25*λ* for *N_T_* = 90 and *N_r_* = 5 and 6.2*λ* for *N_T_* = 216 and *N_r_* = 8. The resolution of minimum spacing in the performance evaluation of Figure 3, Figure 4 and Figure 5 is approximately one wavelength. The response in the performance of the concentric ring array could be improved by increasing the resolution of the minimum spacing in the optimization procedure. This would require more intensive simulations and computational cost. However, the response illustrated in these Figures provide enough information to make a fair comparison among the design cases.

**Figure 6:**
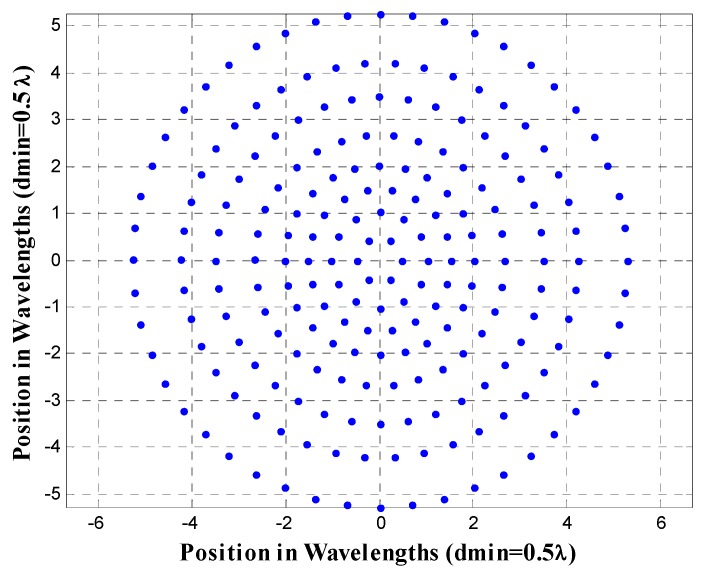
Antenna array configuration of the design case UW-ACRA*_rings_* of *N_T_* = 216 antenna elements with *N_r_* = 8 rings at a frequency *f* = *f*_0_, corresponding to a minimum element spacing of 0.5*λ*.

**Figure 7:**
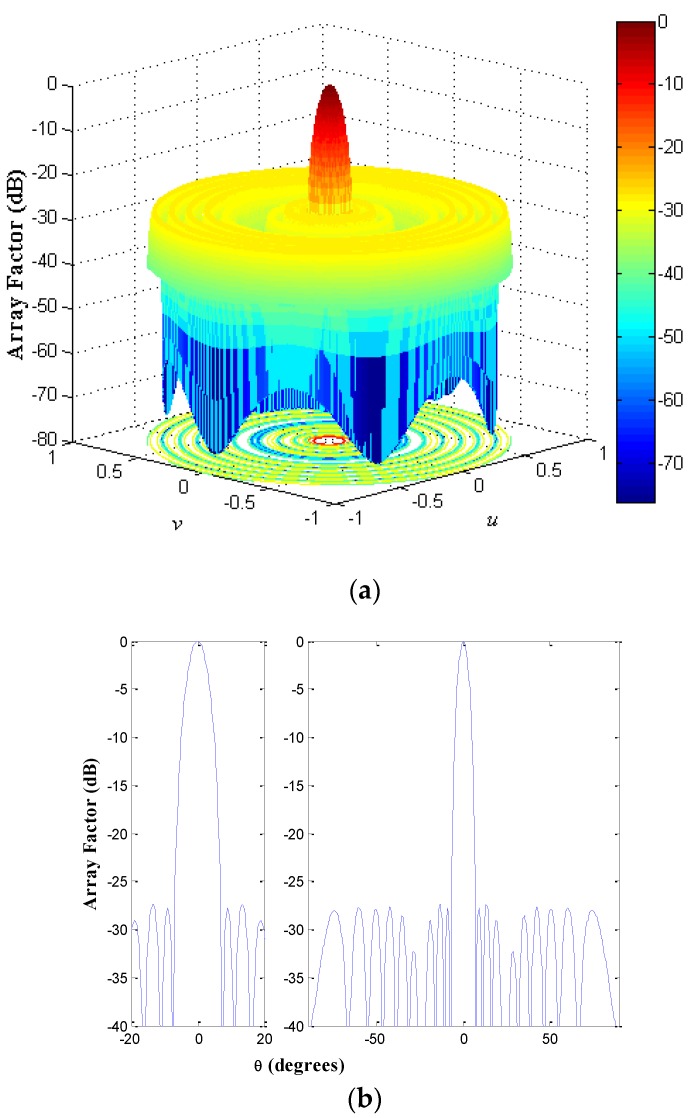
(**a**) Array factor and (**b**) cut of the array factor at *ϕ* = 0° for the antenna array configuration illustrated in Figure 6.

The geometry of the non-uniform concentric rings array for the design case UW-ACRA*_rings_* of *N_T_* = 216 with *N_r_* = 8 rings at a frequency *f* = *f*_0_ (minimum element spacing of 0.5*λ*) is shown in Figure 6. The array factor and a cut of the array factor at *ϕ* = 0° for this antenna array configuration is demonstrated in Figure 7.

**Figure 8:**
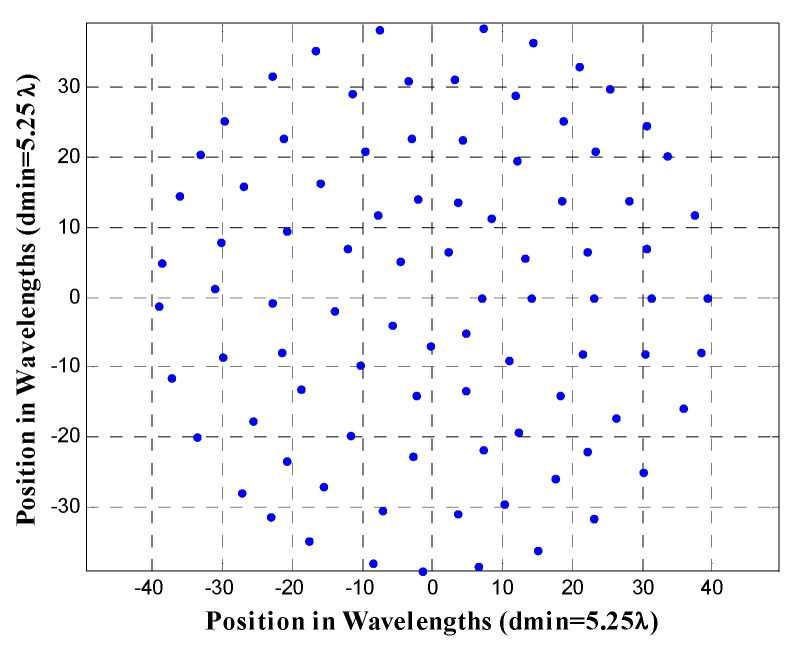
Antenna array configuration of the design case of UW-ACRA*_elements_* of *N_T_* = 90 antenna elements with *N_r_* = 5 rings at a frequency *f* = 10.5*f*_0_, corresponding to a minimum element spacing of 5.25*λ*.

**Figure 9:**
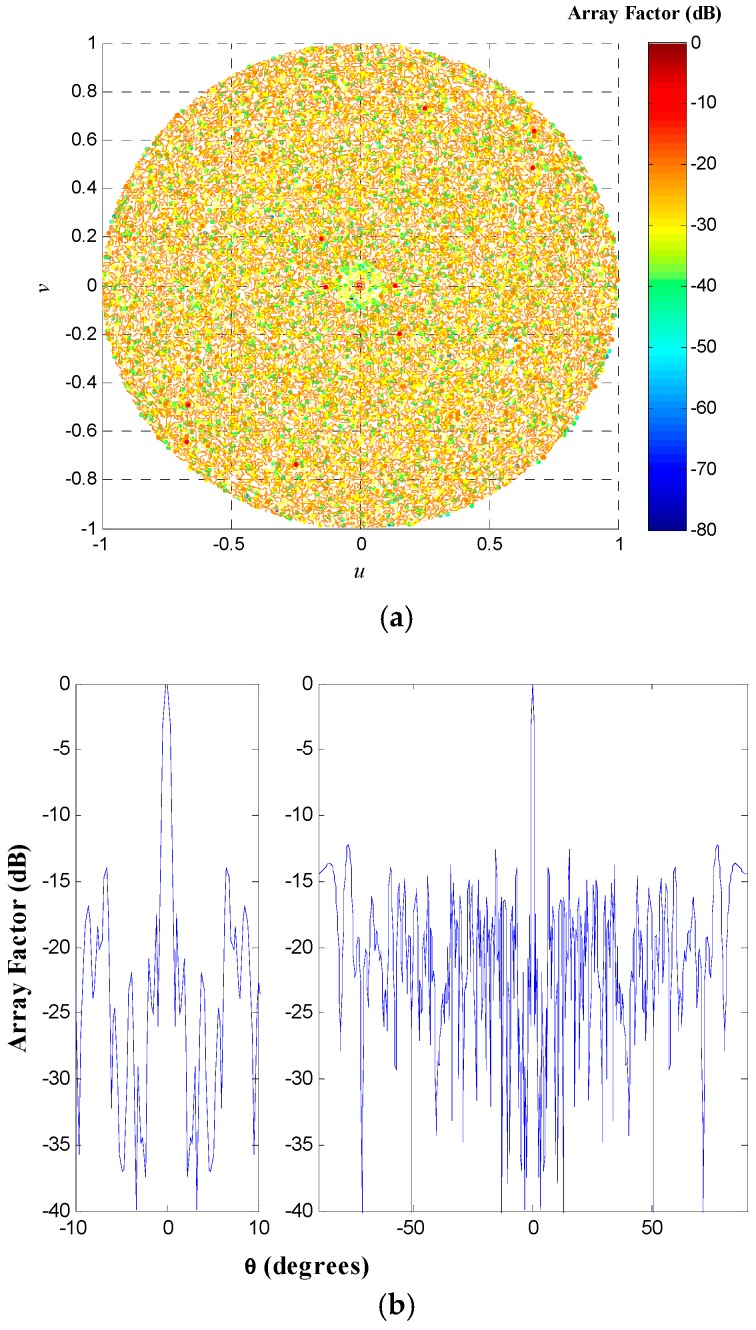
(**a**) Array factor and (**b**) cut of the array factor at *ϕ* = 0° for the antenna array configuration illustrated in Figure 8.

Furthermore, the antenna array configuration of the design case UW-ACRA*_elements_* of *N_T_* = 90 antenna elements with *N_r_* = 5 rings at a frequency *f* = 10.5*f*_0_ (corresponding to a minimum element spacing of 5.25*λ*) is shown in Figure 8. The array factor and a cut of the array factor at *ϕ* = 0° for this antenna array configuration is demonstrated in Figure 9.

Table 1 illustrates a performance summary of the design cases UW-ACRA*_elements_* and UW-ACRA*_rings_*, and a comparative analysis in *PSLL* performance at a frequency *f* = *f*_0_ (*d_min_* = 0.5*λ*) and *f* = 20*f*_0_ (*d_min_* = 10*λ*) with respect to previous works in the literature. As shown in Table 1, the design case UW-ACRA*_rings_* outperforms all design cases when a *d_min_* = 0.5*λ* is set in the array configuration. For high bandwidth ratios the design cases UW-ACRA*_rings_* and UW-ACRA*_elements_* yield a good design trade-off between the number of elements and the *PSLL* performance. In this case, UW-ACRA*_elements_* presents the best compromise between the number of antenna elements and *PSLL* performance for a *d_min_* = 10*λ*.

This paper only considers two design objectives: *SLL* and minimum element spacing (bandwidth). This is because the non-uniform arrays may offer a wider range of frequencies but suffer from a limited ability to predictably control the worst case of peak *SLL*. More design objectives could be considered in the cost function. In that case multi-objective optimization algorithms could be more efficient to search for a Pareto approximation among all the objectives, but that could be the subject of another research manuscript. Furthermore, the results obtained could be integrated with the results of this paper.

## 4. Conclusions

The simulation results demonstrated that for the antenna array configurations of aperiodic concentric rings, the method of *DE* found the element spacing to provide low *PSLL* over an extended bandwidth for the element spacing corresponding to frequencies of *f*_0_ to 20*f*_0_. Both aperiodic geometries present a better *PSLL* performance with respect to the periodic case. The lowest values of *PSLL* (in each design case) are found for *d_min_* = 0.5*λ* with lower values for UW-ACRA*_rings_* with respect to UW-ACRA*_elements_*.

For high bandwidth ratios, the design cases UW-ACRA*_rings_* and UW-ACRA*_elements_* yield a good design trade-off between the number of elements and the *PSLL* performance. However, the design case UW-ACRA*_elements_* presents the best compromise between the number of antenna elements and *PSLL* performance for frequency values greater than 5*f*_0_.

Future studies could investigate the application of multi-objective optimization algorithms to search for a Pareto approximation among all the design objectives. Furthermore, the application of these multi-objective optimization techniques could be extended to other antenna array geometries.

## Figures and Tables

**Table 1:** Performance summary of the design cases UW-ACRA*_elements_* and UW-ACRA*_rings_* and a comparison with respect to previous works.

Array Configuration	Number of Elements	*PSLL* (dB) *d_min_* = 0.5*λ*	*PSLL* (dB) *d_min_* = 10*λ*
Planar array (rotational symmetry) [2]	220	−16.91	−12.05
Planar array (rotational symmetry) [2]	600	−19.2	−16.50
Aperiodic tiling–Penrose optimized [8]	551	−16.49	−6.50
Aperiodic tiling–Danzer optimized [8]	811	−16.01	−10.73
UW-ACRA*_rings_*	90	−24.95	−10.21
UW-ACRA*_rings_*	126	−25.87	−11.16
UW-ACRA*_rings_*	168	−26.59	−12.24
UW-ACRA*_rings_*	216	−27.82	−13.22
UW-ACRA*_elements_*	90	−18.84	−11.20
UW-ACRA*_elements_*	126	−19.91	−12.83
UW-ACRA*_elements_*	168	−20.35	−13.76
UW-ACRA*_elements_*	216	−21.30	−14.66
Periodic Concentric Ring	216	−17.37	−6.30
